# Zebrafish caudal fin model to investigate the role of Cissus quadrangularis, bioceramics, and tendon extracellular matrix scaffolds in bone regeneration

**DOI:** 10.1016/j.jobcr.2025.05.009

**Published:** 2025-06-07

**Authors:** S. Balaji Ganesh, Fharreeha Fathima Anees, Gurumoorthy Kaarthikeyan, Taniya Mary Martin, Meenakshi Sundaram Kishore Kumar, M.I. Sheefaa

**Affiliations:** a Department of Periodontics, Saveetha Dental College and Hospitals, Saveetha Institute of Medical and Technical Sciences (SIMATS), Saveetha University, Chennai, 600077, Tamil Nadu, India; b Zebrafish Facility, Department of Anatomy, Saveetha Institute of Medical and Technical Sciences (SIMATS), Saveetha University, Chennai, 600077, Tamil Nadu, India

**Keywords:** Zebrafish fin regeneration, Cissus quadrangularis, Extracellular matrix, Silver hydroxyapatite, Silver tricalcium phosphate, Carrageenan

## Abstract

**Introduction:**

Periodontal bone regeneration faces several challenges such as incomplete bone-ligament-cementum restoration, unpredictable clinical outcomes and membrane exposure risks. The zebra fish caudal fin model of regeneration could be an excellent model system for mimicking the periodontal regenerative criteria and the present study aimed to explore the potential of innovative composite scaffolds characterized by *C. quadrangularis,* carrageenan, and tendon ECM with silver hydroxyapatite and silver tricalcium phosphate for periodontal bone regeneration by using the zebrafishes as model organisms.

**Materials and methods:**

Zebrafish were subjected to caudal fin amputation, followed by the application of different scaffold groups. The fabricated scaffolds were then crosslinked using glutaraldehyde vapours and stored in a desiccator for future use. The scaffolds were classified into five groups: negative control, PERIO COL- GTR (G1), *Cissus quadrangularis* extract, carrageenan, tendon extracellular matrix (TEM) (Group 2), Group 3 (contained silver hydroxyapatite + Group 2 components), Group 4 (Silver tricalcium phosphate + Group 2 components). Scaffolds characterization was done using FTIR and UV–Vis Spectroscopy. Regeneration was evaluated using microscopically and histologically.

**Results:**

The physical characterization showed the scaffold interaction by spectral differentiation between the groups. Among the groups, Test group 4 demonstrated superior regeneration with enhanced collagen deposition, reduced inflammatory response, and significantly higher growth compared to control.

**Conclusion:**

These findings suggested that group 4 scaffold significantly accelerates zebrafish fin regeneration, making these scaffolds promising candidates for periodontal tissue engineering and regenerative medicine applications.

## Graphical abstract

**Figure:**
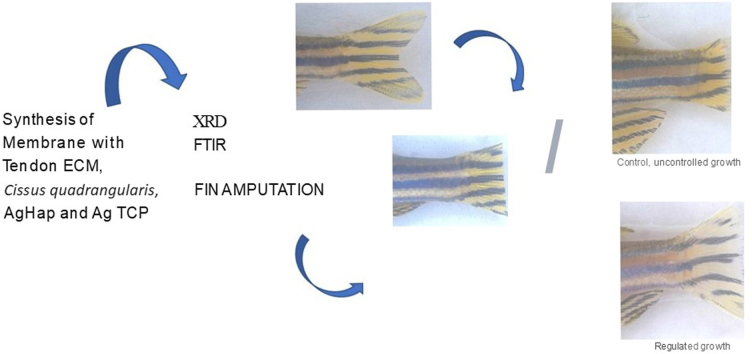

## 1 Introduction

Periodontal guided tissue regeneration is an important method for restoring the periodontal tissue in case of lost tissues such as alveolar bone, periodontal ligament and cementum also. Usage of the barrier membranes facilitate the expansion of the periodontal ligament and associated bone-forming cells for the restoration.^1^ These membranes help in repairing of periodontal defects, alveolar ridge preservation, peri-implantitis also. Besides such advantages, it also faces several drawbacks in regeneration due to lesser extensive area. Application of bioactive products could enhance regeneration by expanding the anti-inflammation and neo-vascularization. The conductive environment for facilitating the bio-signals between the periodontal ligament niche is a prerequisite criterion for achieving the excellent tissue restoration, but, the conventional membranes usually poorly handled the situation like, intra-bony defects, furcation involvement, implant associated bone-loss.^2^ In this scenario, the recent literatures showed that bioactive compounds that can enhance periodontal regeneration by improving cellular responses, angiogenesis, and wound healing acceleration. To mimic this complex tissue niche, several animal models serve to achieve an excellent therapeutic application, among them zebrafish are an important and novel model for the periodontal research.^3^

Zebra fish become a well-recognized animal models for studying the tissue regeneration due to their significant similarity between the human. The regenerative phenomena in caudal fin, retina and spinal cords formation, are seems to be highly organised process on these animals and giving an excellent platform for the evaluation of regenerative medicines.^4^ Recent researches showed that biomaterials such as scaffolds are being evaluated tremendously on zebra fishes. Well organized signalling pathway such as Wnt/β catenin, Fibroblast Growth Factor (FGF), Transforming Growth Factor- β, and Bone Morphogenetic Proteins (BMPs) may enhance the cellular proliferation and differentiation and could be influenced and or inhibited for the drug evaluation purpose on these models.^5^ The wound healing stages such as epithelialization, blastema-formation, cell proliferation and remodelling of tissue organisation are occurring between 7 and 14 days of injury in zebra fishes, as similar to human. Because of genetically and or physiological defects, the wound healing process could be retarded.^6^ Hence, the biomaterials are used to help to provide the structural supports and are acting like bioactive clues to enhance the cellular migration, proliferation and differentiation on wound healing stages. Collagen membranes serve as structure support besides helping in fibroblast attachment, and enhancing angiogenesis.^7^ They also prevent the escape of epithelial cells towards the defected wound sites. PERIO-COL-GTR, is a commercially available collagen membrane, widely used in clinical dentistry particularly on periodontal and bone regeneration. Their biocompatibility and structural integrity support their regenerative efficiency in chronic wounds. Incorporation of bio active compounds and nanoparticles in such kind of supportive membranes are highly enhancing the tissue remodelling by suppression of inflammation.^8^

The phytochemicals are different chemical compounds produced by the plant metabolisms and they are showing effective bioactive nature towards various disease targets. Their profound effects on various cellular processes *viz.,* cell proliferation, cell differentiation and their modulating property on cellular oxidative metabolisms or being recognized exponentially by the scientific era, hence their regenerative potential could be explored to achieve beneficially for regulating the inflammatory and angiogenic criteria in the tissue environment. Previous studies showed that incorporation of phytochemicals into scaffolds could reduce oxidative damage at the wounded sites. Further they had been shown to regulate the key regenerative signalling pathway like Wnt/β catenin and Notch signalling. Among the plants, *Cissus quadrangularis*, are quite common medicinal plants and known as Hadjod, in folkfore medicines. They have excellent anti-inflammatory, antioxidant, and osteogenic potentials.^8^ They are rich in flavonoids, alkaloids and tannins. These plants have been demonstrated to have the ability for accelerating the bone regeneration while moderating the oxidative stress and inflammatory cytokines. Further the recent studies showed that CQ enhances the collagen synthesis, promoting angiogenesis and stimulatingly proliferation of osteoblasts. Hence these plants are bio active incorporations for tissue engineered scaffolds.^9^ Carrageenan, from *Kappaphycus alvarezii,* gained attention due to its versatile biocompatibility, biodegradability and gelling properties. It has been extensively used in hydrogel formulation and scaffold composites for promoting the wound healing properties. Further, it showed to exhibit the controlled delivery of the bioactive compounds in to the wounded sites.^10^ Recent studies showed that this kind of this kind of scuffles facilitate the cellular attachment proliferation and matrix remodelling. In addition, they had been showed to have better anti-inflammatory, and immune-modulatory properties that help in reducing the inflammatory responses for making an ideal situation for chronic wound healing and bone regeneration.^11^ Tendon extracellular matrix (Tendon ECM) helps as a natural reservoir for collagen, glycosaminoglycans and various growth factors on wound healing purposes. Decellularized Tendon ECM retains its three-dimensional structure and promoting the cellular attachment, migration, differentiation and collagen deposition. Bioceramics have been shown to have biocompatibility and osteoconductivity. Further those bioceramics are also known as “Ion releasing reservoirs” that can very helpful in calcium and phosphate ions for enhancing the mineralization and angiogenesis at the wounded sites. In addition to the bioceramics also influence the cellular adhesion, migration and proliferation positively. Silver hydroxyapatite (Ag- HAp) and Silver tricalcium phosphate (Ag- TCP) have excellent anti-microbial, osteo-inductive, and regenerative properties.^12^ For aiding the regenerative potential collagen compositions such as tendon ECM are used to provide a mechanical strength as a well-organized structure called biological scaffolds. Incorporation of these composite scaffolds could induce cellular recruitment, differentiation and matrix deposition in the tissue regenerative nicke. Hence in the zebrafish caudal fin model these phytochemicals, bioceramics and tendon ECM would facilitate the organization of regenerative cells for the structural restoration.^10^, ^11^, ^12^

Silver nano particles are widely used for their antimicrobial activity against wide variety of micro real pathogens, to inhibit the infection and biofilm formation. HAp, are naturally existing mineral forms that could induce the osteo-conduction for regeneration and mineralization. On doping with silver ions gain the antimicrobial properties besides with the osteo-induction.^13^ Zebrafish caudal fin amputation model is inexpensive, morally acceptable and it can be used as a regenerative animal model for screening biomaterials used for tissue engineering at an early stage. The zebra fish caudal fin model of regeneration could be an excellent model system for mimicking the periodontal bone regenerative criteria and the studies related to the effect of combining phytochemicals, bioceramics and tendon ECM on caudal fin regeneration are scarcely available, hencethe present study aimed to explore the potential of innovative composite scaffolds characterized by *C. quadrangularis,* carrageenan, and tendon ECM with silver hydroxyapatite and silver tricalcium phosphate for periodontal bone regeneration by using the zebrafishe as model organisms.^7^, ^8^, ^9^, ^11^, ^12^, ^14^ As far as we know this is the first of its kind and the findings from this study can provide valuable insights into the future therapeutics for tissue regeneration.

## 2 Materials and methods

Fresh *Cissus quadrangularis* stems were collected, washed and shade-dried. The dried stems were then powdered and extracted using a Soxhlet apparatus with 95 % ethanol for 48 h. The extract was filtered and concentrated using a rotary evaporator at 40 °C under reduced pressure. The resulting crude extract was stored at −20 °C until further use.

### 2.1 Synthesis of bioceramics

Silver hydroxyapatite (HA) and silver tricalcium phosphate (TCP) bioceramics were synthesized using the sol-gel technique. HA was prepared by combining calcium nitrate and ammonium phosphate in a 1.67:1 M ratio, with the pH adjusted to 10 using ammonia. The mixture was then aged for 24 h and subjected to calcination at 800 °C. Meanwhile, TCP was synthesized through a silica gel-based approach, utilizing calcium and phosphate precursors.^14^

### 2.2 Extraction and processing of tendon ECM (TEM)

The porcine/Achilles tendon was decellularized using a detergent-based approach involving 1 % SDS and 0.1 % Triton X-100, followed by thorough washing with PBS. The resulting extracellular matrix (ECM) was freeze-dried, pulverized into a fine powder, and subsequently reconstituted into a hydrogel.^14^

### 2.3 Scaffold fabrication

Scaffolds were developed by integrating Cissus quadrangularis extract, carrageenan, and tendon ECM hydrogel using the electrospinning technique, with polycaprolactone (PCL) as the base polymer to ensure mechanical stability. A 10 % w/w PCL solution was blended with varying ratios of carrageenan, hydroxyapatite (HA), and tricalcium phosphate (TCP), along with ECM and plant extract, followed by electrospinning under optimized conditions (voltage: 15 kV, flow rate: 1.2 mL/h). The fabricated scaffolds were then crosslinked using glutaraldehyde vapours and stored in a desiccator for future use. The scaffolds were classified into four groups: Group 1 served as the positive control (PERIO COL), Group 2 contained Cissus quadrangularis extract, carrageenan, and tendon ECM, Group 3 incorporated silver hydroxyapatite along with Cissus quadrangularis extract, carrageenan, and tendon ECM, while Group 4 included silver tricalcium phosphate in combination with *Cissus quadrangularis* extract, carrageenan, and tendon ECM. Negative control group was not treated with any scaffold.

### 2.4 Characterization techniques

Fourier Transform Infrared Spectroscopy (FTIR) (PerkinElmer, USA) was carried out to identify functional groups and confirm molecular interactions among scaffold components, with spectra recorded within the range of 4000–400 cm^−1^. To assess the optical characteristics and verify the effective integration of the silver-based compound into the corresponding membranes, a UV–Vis spectroscopy examination (PerkinElmer, Lambda 365+, USA) was conducted.^14^

### 2.5 Zebrafish husbandry and fin regeneration evaluation

All zebrafish experiments were conducted following approval from the Institutional Animal Ethical Committee of Saveetha Dental College and Hospitals, Saveetha Institute of Medical and Technical Sciences (SIMATS) (Approval No.: BRULAC/SDCH/SIMATS/IAEC/06–2023/15). The study adhered to established guidelines for the care and use of laboratory animals. Wild-type zebrafish (Danio rerio) were housed in a recirculating aquaculture system under controlled conditions, maintaining a temperature of 28 °C with a 14:10 light-dark cycle.

The adult zebra fish (*Danio rerio,* AB type, 3–6 months old) were corrupted from the local shops and acclaimed 15 days with 15 L of tanks in laboratory condition. The caudal fins were amputated using a scalpel, and regeneration was monitored until specific time points at 33 °C. The fish were then anesthetized using 0.016 % tricaine methanesulfonate, and the regenerated tissues were collected for further analysis. The fish were then anesthetized using 0.016 % tricaine methanesulfonate, and the caudal fin were amputated carefully with the scapel. A cut was made parallelly to the dorsal and ventral axis. Image was taken using the stereomicroscope (Leica M205C) and considered as control (Day 1). Image J software was used to find out the area of the amputated and regenerated fins by adopting the manufactures instructions. The amputated fish were incubated with the respective scaffolds as follow. The group 1 consist of positive control group treated with PERIO COL-GTR (conventional collagen membrane). Group 2 scaffold contained *C. quadrangularis,* carrageenan, and Tendon extracellular matrix. Group 3 scaffold contained silver hydroxyapatite, *C. quadrangularis,* carrageenan, and Tendon extracellular matrix. The group 4 contained scaffold of silver tricalcium phosphate, *C. quadrangularis,* carrageenan, and Tendon extracellular matrix. Negative control group was not treated with any scaffolds. Further, the images were taken as 7 and 14th post amputated day. During the experiment the fish were fed twice a day with the same quantity of commercially available food and the respective membrane were replaced daily. The regeneration efficiency (%) was calculated by using the following formula, regeneration efficiency (%) = (Regenerated fin area/Fin area at control) x 100. The experiments were done in triplicate and the data were represented as mean ± standard deviation.^14^

### 2.6 Histological analysis

Amputated fins were preserved in 4 % paraformaldehyde, embedded in paraffin, and sectioned for microscopic examination. To assess tissue regeneration, the sections were stained using Hematoxylin and Eosin (H&E) staining.^14^

### 2.7 Statistical analysis

All experiments were conducted in triplicates, and the data were analyzed using GraphPad Prism software. Statistical significance was evaluated using two-way ANOVA, with p < 0.05 considered statistically significant.^10^, ^14^

## 3 Results

### 3.1 UV analysis

**Fig. 1:**
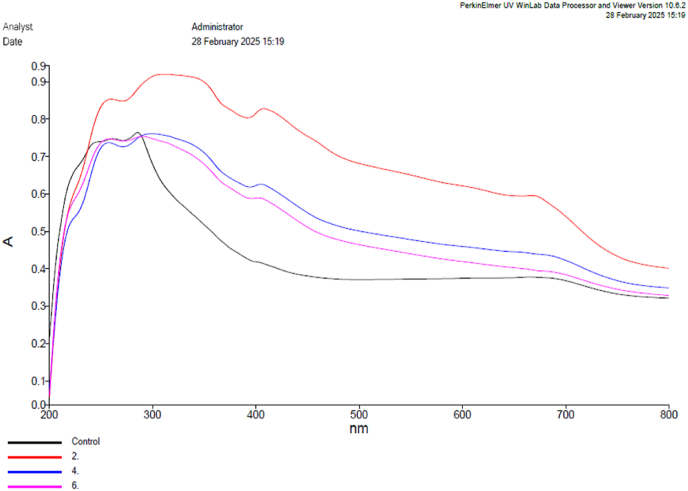
UV spectral analysis of the synthesized scaffold vs Control.

UV–Vis spectroscopy analysis was done to evaluate the optical properties and confirmation of the successful incorporation of silver-based compound in to the respective membranes. Group 1, 2 and 3, exhibited listing pattern characteristics by the structural modification and molecular interactions within the scaffolds. Group two show repeat around 280–300 nm indicated the π- π∗ of phenolic, meanwhile, Peak around 322–350 nm showed the presence of amide bonds and hydroxyl groups from ECM and polysaccharide matrix. Group 3 contained silver hydroxyapatite with the primary absorption on 310–330 nm. This suggested that a shift in electronic confirmation due to interaction between the ECM proteins and carrageenan’ sulphate groups. Due to the incorporation of phosphate and calcium ions within the scaffold matrix, group 4 showed characteristic peak at 320–340 nm, this result suggested the potential ionic interactions that lead to scuffles electronic configuration. UV analysis revealed the successful incorporation and synthesis of scuffled matrix (Fig. 1).

### 3.2 FTIR analysis

**Fig. 2:**
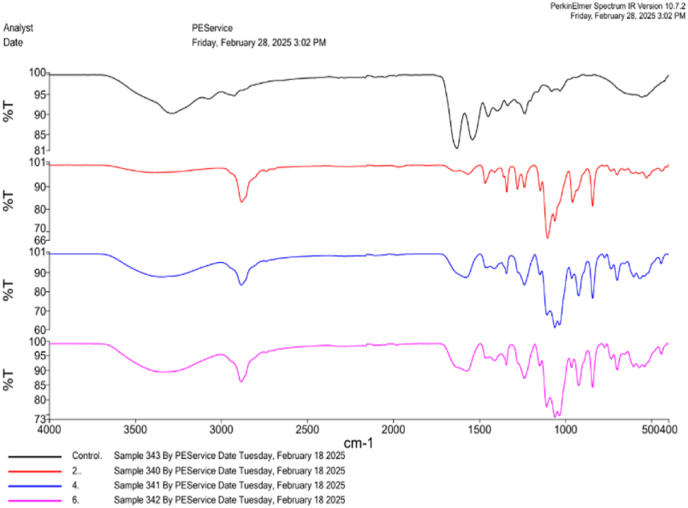
FTIR spectral analysis of the synthesized scaffold vs Control.

The FTIR spectral analysis compared the Group 1 (PERIO COL), group 2 with *Cissus quadrangularis,* carrageenan, and tendon ECM-based scaffold; and Group 3 with *silver hydroxyapatite, Cissus quadrangularis,* carrageenan and tendon ECM-based scaffold; and Group 4 with silver tricalcium phosphate, *Cissus quadrangularis,* carrageenan and tendon ECM-based scaffold to evaluate the structural modifications (Fig. 2). The transmittance (%T) values across the wave number ranged from 4000 to 400 cm^−1^ revealed distinct spectral variation between the groups, indicating molecular interactions and successful incorporation of silver based compounds. The overall transmittance differences between the groups suggested that silver hydroxyapatite and silver tricalcium phosphate influenced the ECM scaffold and polysaccharide matrix modify hydrogen bonding and positive interactions. Test group 3 demonstrated greater structural stability due to hydroxyapatite affinity for ECM proteins whereas test group 4 exhibited more ionic interactions with Carrageenan and ECM components. These findings confirmed that silver containing compounds successfully integrated into the scaffold while inducing distinct molecular alterations which could affect the air biomechanical properties bio activity and potential for tissue engineering purposes.

### 3.3 Fin regeneration analysis: tissue regeneration and growth

The zebra fish fin regeneration experiment showed the growth of fine-tuned patterns between the control and the tested materials due to the regulation of the signalling pathways. Though the noticeable fin growth in control was seen, the bifurcation was absent compared to the tested materials. This result suggested the controls achieved the disorganized tissue remodelling and incomplete pattern of morphogens. In contrast, group 4 treatment revealed the presence of superior fin regeneration while, with a well-defined bifurcation, and structural restoration. Group 1, 2 and 3 also showed increased fin length after amputation over 7 and 14 days, but their growth was partial and inconsistent on tissue organization. This result indicated that the regeneration was sub optimal, and failed to induce the structural precision during the regeneration period. Further, molecular level analysis should be done for the confirmation.

**Fig. 3:**
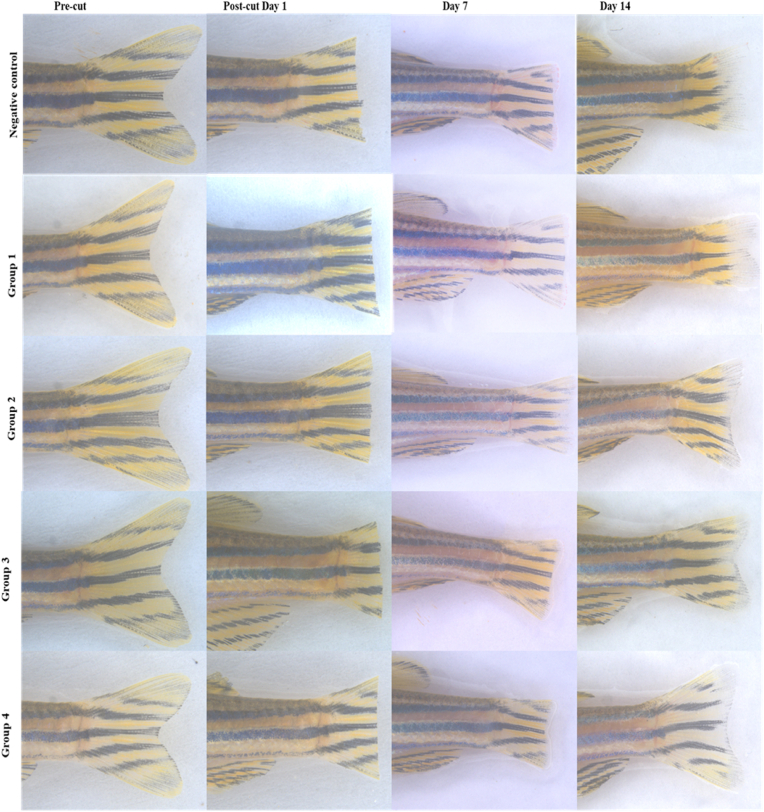
**Effect of synthesized and control scaffolds on Amputated Fin of the zebra fishes.** The amputated zebrafish fins treated with, negative control, PERIO COL- GTR (G1), *Cissus quadrangularis* extract, carrageenan, tendon extracellular matrix (TEM) (Group 2), Group 3 (contained silver hydroxyapatite + Group 2 components), Group 4 (Silver tricalcium phosphate + Group 2 components).

**Fig. 4:**
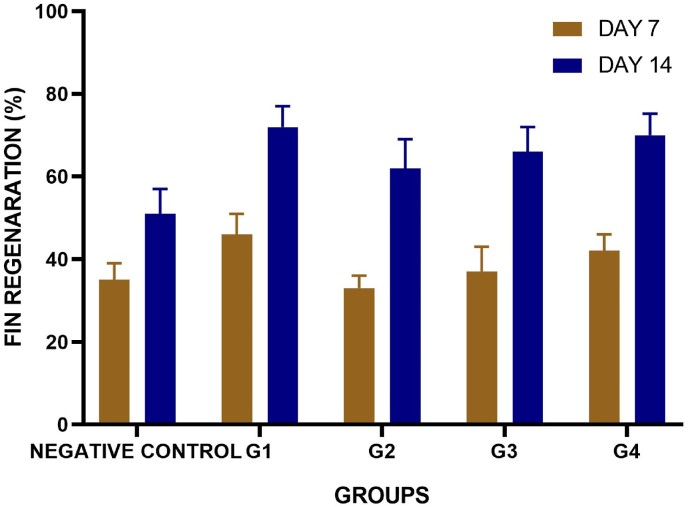
**Fin Regeneration (%) of amputated zebrafish treated with synthesized and control scaffolds.** negative control, PERIO COL- GTR (G1), *Cissus quadrangularis* extract, carrageenan, tendon extracellular matrix (TEM) (Group 2), Group 3 (contained silver hydroxyapatite + Group 2 components), Group 4 (Silver tricalcium phosphate + Group 2 components). The independent three experimental data were expressed as mean ± SD (n = 6/group, Two way ANOVA, p = 0.001 for all groups).

The initial and final fin length ware recorded and the fin growth (%) over the time was plotted and represented in Fig. 3. The plotted data showed fin growth initially, disorganized laterally. The group 4 showed better performance than the other group (Fig. 4). The regeneration efficiency was found to be statistically significant using two way ANOVA test (p = 0.001)

### 3.4 Histology analysis

**Fig. 5:**
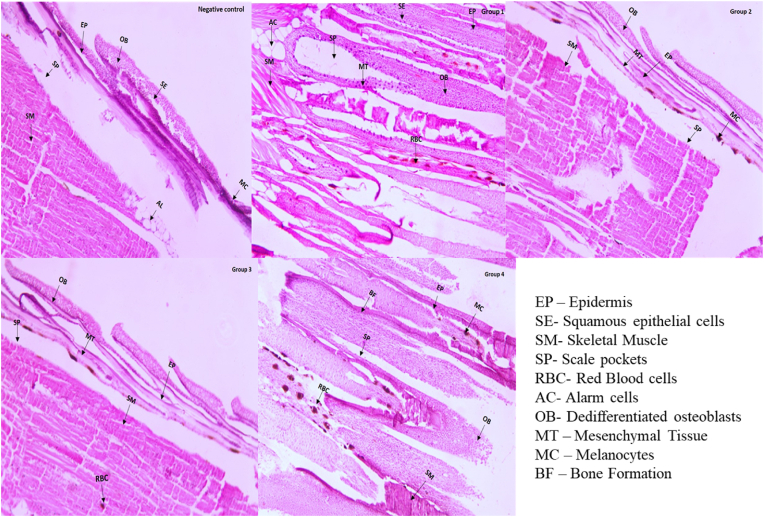
**Histological analysis of amputated fin of zebrafish tissue treated with synthesized and control scaffolds.** Negative control, PERIO COL- GTR (G1), *Cissus quadrangularis* extract, carrageenan, tendon extracellular matrix (TEM) (Group 2), Group 3 (contained silver hydroxyapatite + Group 2 components), Group 4 (Silver tricalcium phosphate + Group 2 components).

Compared to the tissue regeneration across five experimental groups, group 4 demonstrated most of the outcomes compared to the others. Group 4 exhibited bone formation characterised by increased osteoblast activity and matrix deposition, indicated by efficient tissue remodelling. The presence of differentiated epithelial layer in group 4, further suggested active proliferation and differentiation contributing to superior regeneration. The mesenchymal tissue (MT) showed the prominent tissue repair epidermis and squamous epithelial cells indicated effective stem cell requirement and pronunciation which played crucial role in enhancing issue architecture. The presence of Scale Pockets (SP) indicated the remodelling and regeneration during the treatment and alarm cells (AC) and red blood cells (RBC) also sign of regulated inflammatory response and vascularization. In groups 2, 3 and positive control, slower and lesser regeneration was indicated by prolonged tissue processes. Further the lesser density of melanocytes (MC), suggested the reduced growth. Bone formation (BF) was minimal due to lesser differentiated osteoblasts (OB) in these groups (Fig. 5). The growth of caudal in regeneration was monitored using stereo microscopy at one, 7 and 14 days post amputation (dpa). The subsequent images revealed that gradual increase of the fin tissue at all groups, while a significant enhancement was found between the treated groups than the control group. Group one the untreated group showed a slow and incomplete regeneration in which minimum migration was see at day 7 and 14. The group 1 treated with a conventional collagen membrane showed moderate regeneration with the significant epithelial closure and characteristically partial fin regeneration on day 7 and 14. Group 4 show enhanced fin growth and length compared to the groups two and three. Group 4 exhibited the most significant film regeneration by near completion of fin tissue restoration on Day 14. The group 3 showed notable tissue regeneration higher than group two scaffold but lesser than group four scaffold (Fig. 5).

## 4 Discussion

Periodontal bone regeneration is a complex biological process aimed at restoring the structural and functional integrity of alveolar bone that has been lost due to periodontitis, trauma, or surgical interventions. Unlike conventional treatments that primarily focus on halting disease progression, true periodontal regeneration seeks to reconstruct lost bone, periodontal ligament, and cementum, thereby restoring the complete periodontal architecture. Various regenerative strategies, including the use of growth factors, stem cells, and biomaterials, have been explored, with scaffold-based approaches emerging as one of the most promising solutions.^15^

Scaffolds serve a crucial function in periodontal bone regeneration by providing a temporary three-dimensional matrix that supports cellular attachment, proliferation, and differentiation. These biomaterials act as structural frameworks that facilitate osteoconduction while promoting the deposition of new bone. An ideal scaffold must be biocompatible, biodegradable, and mechanically stable to closely mimic the properties of native bone while allowing for vascularization and integration with surrounding tissues. The composition of the scaffold is critical, as it determines its ability to support cell migration, growth factor retention, and extracellular matrix deposition, all of which contribute to successful bone regeneration.^12^, ^14^, ^15^, ^16^

Our research focuses on developing and analysing novel scaffolds that incorporate Cissus quadrangularis extract, carrageenan, and tendon extracellular matrix (TEM), along with bioactive ceramics such as silver hydroxyapatite (Ag-HA) and silver tricalcium phosphate (Ag-TCP), to enhance the regenerative potential of periodontal bone tissues. The first type of scaffold in our study consists of Cissus quadrangularis extract, carrageenan, and TEM. Cissus quadrangularis is a well-known medicinal plant that has demonstrated osteogenic potential by stimulating osteoblast differentiation and enhancing bone healing.^17^ Carrageenan, a naturally derived polysaccharide, improves the mechanical strength and structural integrity of the scaffold while also promoting a favourable microenvironment for cell growth. The addition of TEM provides essential extracellular matrix components that facilitate cellular interactions, encourage cell adhesion, and promote tissue remodelling.^16^, ^17^, ^18^

To further enhance the regenerative potential of this scaffold, the second variation integrates silver hydroxyapatite (Ag-HA) with the base components. Hydroxyapatite, a widely used bone substitute material, is highly bioactive due to its structural similarity to the mineral phase of bone. It provides excellent osteoconductive, which supports new bone growth and integration with surrounding tissues. The incorporation of silver into hydroxyapatite introduces antimicrobial properties, thereby reducing the risk of infection, which is a common challenge in bone grafting and tissue regeneration. This modified scaffold is expected to provide enhanced osteogenic support while ensuring a sterile environment for optimal healing.^14^, ^15^, ^18^, ^19^

Building upon this composition, the fourth scaffold variation includes silver tricalcium phosphate (Ag-TCP) in addition to the previously mentioned components. Tricalcium phosphate is another well-established bio ceramic known for its ability to promote faster bone remodelling and resorption, thereby accelerating the natural healing process. The presence of silver in tricalcium phosphate further reinforces antimicrobial protection, making this scaffold highly suitable for clinical applications where infection control is crucial. By integrating Ag-TCP into the scaffold design, the goal is to enhance both the bioactivity and biodegradability of the material, ensuring that it gradually dissolves and is replaced by newly formed bone over time.^20^

The current study comparatively evaluated the fin regeneration potential of the PERIO COL GTR, a conventional collagen membrane, alongside novel composite scaffolds incorporating Cissus quadrangularis, carrageenan, and tendon extracellular matrix, with additional incorporation of silver-hydroxyapatite and tricalcium phosphate using multiple analysis such as UV, FTIR, histology. The results showed that structural and biochemical alterations were superiorly altered the regenerative efficacy of the amputated fins among the experimental groups. The spectroscopic analysis provided characteristic structural integrity and molecular interaction taken place by incorporation of silver nanoparticles particularly on group 3 and 4. FTIR peaks confirmed the presence of bioactive compounds based on the peak correspondence further XRD analysis provided a validated results on crystallinity of the incorporated bio ceramic components in the respective synthesized membranes. UV analysis conformed the stability and molecular interactions of such bioactive compounds with the membranes based on spectral absorbing characters. Group 4 scaffold showed controlled and structured regrowth compared to other groups that had several inconsistencies including unregulated outgrowths and options of bifurcation.^8^, ^9^, ^10^, ^12^, ^13^, ^14^, ^17^

Histological insights provided the characteristics well organized cellular architecture with significant bone formation, mesenchymal tissue formation and red blood cell infiltration. In contrast, except group four other groups also showed regeneration, but inadequate osteoblast differentiation and suboptimal regenerative potentials as confirmed by the histological analysis. These results are concordant with the recent studies highlighted the role of bioactive ceramics and ECM based scaffolds could accelerate the cellular reorganization and regeneration effectively.^21^

In addition to the present study also resonate the composition of the tricalcium phosphate and biopolymers could enhance the osteogenesis and fin regrowth. Similar to the present study previously the literature showed that collaboration of ECM based materials significantly promoting the tissue development by the mesenchymal interactions.^14^, ^15^, ^17^, ^18^, ^20^, ^21^ This study contributed significant insights that the bioactive scaffolds with tendon ECM and tricalcium phosphate could strongly enhance the fin regeneration on zebrafish models and laid a foundation for the future translational research, eventhough it is a preliminary analysisMeanwhile, further molecular level analysis should accompany with this results in future to facilitate the deeper understanding on the underlining fin regenerative potential of group 4 components.

## 5 Conclusion

The composite scaffolds contained with silver tricalcium phosphate, *C. quadrangularis*, carrageenan, and Tendon extracellular matrix (Group 4) exhibited efficient regenerating potential over hydroxyapatite containing combination (Group 3) on amputated zebrafish fins. The physical characterization using UV and FTIR showed the scaffold interaction at molecular levels by spectral differentiation between the groups. Histological analyses showed that Group 4 exhibited organised tissue regeneration than unorganized growth pattern found on other groups including both control groups. These findings aligned with the recent literature that highlighting the importance of ECM based materials in tissue repair. Further studies should focus on therapeutic application of such materials in translational research for the welfare of humankind.

## Patient's/guardian's consent

The authors declare that the above-mentioned manuscript has no need of Patient's/Guardian's consent since, it is a non-clinical research manuscript.

## Declaration of ethical clearance

All zebrafish experiments were conducted following approval from the Institutional Animal Ethical Committee of Saveetha Dental College and Hospitals, Saveetha Institute of Medical and Technical Sciences (SIMATS) (Approval No.: BRULAC/SDCH/SIMATS/IAEC/06–2023/15).

## Declaration of competing interest

The authors declare that they have no known competing financial interests or personal relationships that could have appeared to influence the work reported in this paper.
